# N6-(2-hydroxyethyl)-Adenosine Induces Apoptosis via ER Stress and Autophagy of Gastric Carcinoma Cells In Vitro and In Vivo

**DOI:** 10.3390/ijms21165815

**Published:** 2020-08-13

**Authors:** Hongqing Xie, Xiaotong Li, Weiwei Yang, Liping Yu, Xiasen Jiang, Yajie Chen, Zhangfei Shen, Conghui Li, Meier Gu, Liangen Shi

**Affiliations:** 1 College of Animal Sciences, Zhejiang University, Hangzhou 310058, China; xiehongqing@zju.edu.cn (H.X.); lixiaotong@zju.edu.cn (X.L.); jxsen@zju.edu.cn (X.J.); yajiesw@126.com (Y.C.); szf@zju.edu.cn (Z.S.); lch941114@163.com (C.L.); 2 Laboratory Animal Center, Hangzhou Normal University, Hangzhou 311121, China; dwzxyww@163.com (W.Y.); ylp1975@hznu.edu.cn (L.Y.)

**Keywords:** N6-(2-Hydroxyethyl)-adenosine, apoptosis, endoplasmic reticulum stress, gastric carcinoma, autophagy

## Abstract

Gastric cancer is the most common malignant tumor of the digestive tract and is great challenge in clinical treatment. N6-(2-Hydroxyethyl)-adenosine (HEA), widely present in various fungi, is a natural adenosine derivative with many biological and pharmacological activities. Here, we assessed the antineoplastic effect of HEA on gastric carcinoma. HEA exerted cytotoxic effects against gastric carcinoma cells (SGC-7901 and AGS) in a dose and time-dependent manner. Additionally, we found that HEA induced reactive oxygen species production and mitochondrial membrane potential depolarization. Moreover, it could trigger caspase-dependent apoptosis, promoting intracellular Ca^2+^-related endoplasmic reticulum (ER) stress and autophagy. On the other hand, HEA could significantly inhibit the growth of transplanted tumors in nude mice and induce apoptosis of tumor tissues cells in vivo. In conclusion, HEA induced apoptosis of gastric carcinoma cells in vitro and in vivo, demonstrating that HEA is a potential chemotherapeutic agent for gastric carcinoma.

## 1. Introduction

Gastric cancer is a malignant tumor originating from gastric mucosal epithelium; it is the fifth most common cancer (with 951,000 new cases per year) and has the third lowest survival rate (754,000 deaths each year worldwide, of which more than 70% occur in developing countries) [1,2]. Surgery is the main treatment option for gastric carcinoma, and adjuvant therapy should also be considered. The five-year survival rate of patients receiving chemotherapy was found to improve by 11% after surgery [3]. In Europe, the preferred treatment scheme for gastric carcinoma is perioperative chemotherapy with epirubicin, cisplatin, or fluorouracil (5-FU) [4]. However, chemoradiotherapy-associated acute toxicity should also be given attention. To study the efficacy of adjuvant therapy after surgery, a controlled trial involving 556 patients with gastric carcinoma revealed that chemoradiotherapy could remarkably improve survival rate and prolong survival time; however, three patients (1%) died from the toxicity of chemoradiotherapy, 41% of the chemoradiotherapy group had grade 3 toxicity, and 32% had grade 4 toxic effects [3]. Therefore, it is necessary to search for effective chemotherapeutic agents for gastric carcinoma with less side effects.

**Figure 1:**
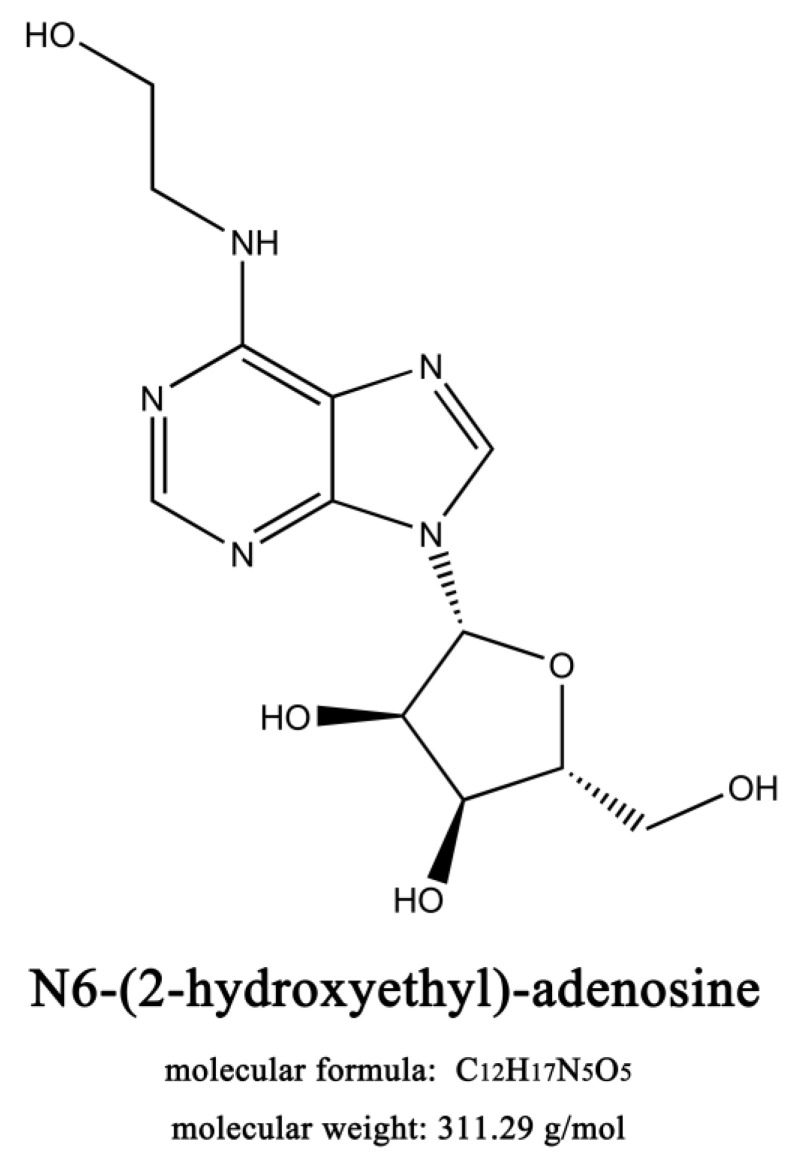
Chemical structure of HEA. The molecular formula of HEA is C_12_H_17_N_5_O_5_, and the molecular weight is 311.29 g/mol.

Various fungi of *Cordyceps* species have anticancer activities [5]. As one of the main biological components of *Cordyceps* species, N6-(2-Hydroxyethyl)-adenosine (HEA) was first isolated from *Cordyceps* and *Isaria* species in 1983 (structure is shown in Figure 1). The separation and preparation of HEA have been reported [6,7]. Pharmacological studies found that HEA possesses antihyperglycemic, kidney protective, antioxidant, sedative, anti-inflammatory, and myocardium protective effects and interacts with human serum albumin [8,9,10,11,12].

The identification of active ingredients and the verification of the pharmacological actions of natural products are the current research trends. Previous studies have shown that the ethanolic extract of *C. cicadae* (EEC) has antitumor effects in vitro, and HEA is one of the main components of EEC [13]. In recent years, a few studies have investigated the antitumor effects of HEA, but its efficacy and mechanism are still unclear [14]. Chronic inflammation is one of the precipitating factors of tumorigenesis. Additionally, the inhibition of NF-κB signal can lead to apoptosis of transformed hepatocytes and failure to develop into hepatocellular cancer [15]. Moreover, TGF-β protein induces apoptosis and cell cycle arrest in the early stage of tumorigenesis. However, with the development of tumors, increased levels of TGF-β promote tumorigenesis by stimulating immune system migration, invasion, angiogenesis, and escape [16]. HEA can regulate the NF-κB and TGF-β signaling pathways in vivo and in vitro and inhibit the production of IL-1β, TNF-α, prostaglandin E_2_, and cyclooxygenase-2 to exert anti-inflammatory effects [10,17]. These findings suggest that HEA may be an ideal candidate for cancer chemotherapy. It is worthwhile to study the anticancer potentiality of HEA.

In this work, we demonstrated that HEA inhibited the proliferation of gastric carcinoma in vitro and in vivo for the first time. Mechanism studies showed that HEA induced ER stress and autophagy-mediated apoptosis in vitro. HEA inhibited the growth of SGC-7901-infected tumors and induced the apoptosis of tumor tissues in nude mice. These results suggested that HEA may become a chemotherapeutic agent for gastric carcinoma.

## 2. Results

### 2.1. HEA Induced Cytotoxicity Effects in Gastric Carcinoma Cells

**Figure 2:**
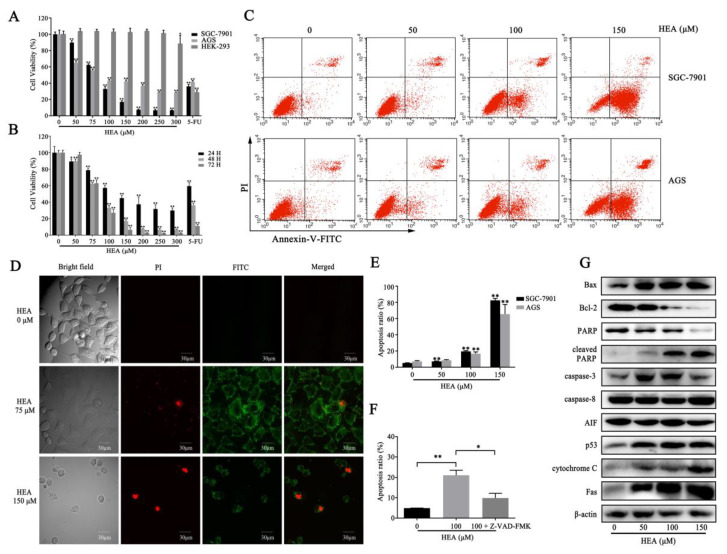
HEA induced inhibitory effects in gastric carcinoma cells. (**A**) Estimation of cytotoxicity using CCK-8 assay with various doses of HEA in SGC-7901, AGS, and HEK293 cells. The cells (SGC-7901, AGS, and HEK293) were treated with 0, 50, 75, 100, 150, 200, 250, and 300 μM HEA and 20 μg·mL^−1^ 5-FU for 48 h and were then detected using CCK-8 assay, with five repeats per group. (**B**) Evaluation of cytotoxicity using CCK-8 assay with various doses of HEA at different treatment times in SGC-7901 cells. SGC-7901 cells were treated with HEA and 5-FU for 24, 48, and 72 h and were then evaluated for cell viability, with five repeats per group. (**C**) HEA promoted apoptosis of gastric carcinoma SGC-7901 and AGS cells. SGC-7901 and AGS cells were incubated with HEA (0, 50, 100, and 150 μM) for 48 h, and apoptosis level was detected via Annexin V-FITC/PI assay. (**D**) Apoptosis level changes in SGC-7901 cells detected by a laser confocal microscope after Annexin V-FITC/PI staining. (**E**) Columns for SGC-7901 and AGS apoptosis levels when incubated with HEA (0, 50, 100, and 150 μM). (**F**) Columns for apoptotic SGC-7901 cells incubated with vehicle, HEA (100 μM), and HEA (100 μM) + Z-VAD-FMK (20 μM). (**G**) Effect of HEA on protein apoptosis in relation to SGC-7901 cells. Cells were treated with HEA (0, 50, 100, and 150 μM), and proteins were extracted for Western blot analysis. ** *p* < 0.01, * *p* < 0.05 compared with 0 μM HEA group, three repeats per group.

Previous studies have shown that the EEC has a lower IC_50_ value for gastric carcinoma SGC-7901 cells than for lung cancer H1299 cells, hepatocellular carcinoma HepG2 cells, lung carcinoma A549 cells, and cervical cancer HeLa cells [13]. To verify whether HEA is the major anti-gastric cancer compound in EEC, we used the gastric cancer cell lines SGC-7901 and AGS as the target cancer cell lines, and used HEK293 cells used in EEC research as the normal cell line. We investigated the toxicity of HEA in gastric carcinoma cell lines SGC-7901 and AGS, and human embryonic kidney cell line HEK293. The results showed that HEA exerted cytotoxic effects against SGC-7901 and AGS cells in a dose and time-dependent manner (Figure 2A,B and Figure S1). We calculated that the IC_50_ value of HEA against SGC-7901 cells was 86.66 μM, and the IC_50_ value of HEA against AGS cells was 94.46 μM. These findings suggest that HEA exerted cytotoxic effects against gastric carcinoma cells. By contrast, HEA had no significant toxicity to HEK293 cells until its dose reached 250 μΜ, demonstrating the toxicity of HEA for gastric carcinoma cells over normal cells (Figure 2A).

### 2.2. HEA Promoted Apoptosis in Gastric Carcinoma SGC-7901 and AGS Cells

To determine the apoptotic effect induced by HEA, we performed an Annexin V-FITC/PI assay using flow cytometry, and a laser confocal microscope was used for observation after Annexin V/PI staining. The total percentages of apoptotic SGC-7901 cells were 5.22% ± 0.07% for the control group, 7.17% ± 0.20% for the HEA 50 μM group, 19.58% ± 0.79% for the HEA 100 μM group, and 82.51% ± 6.09% for the HEA 150 μM group. The total percentages of apoptotic AGS cells were 7.26% ± 0.89% for the control group, 8.59% ± 0.76% for the HEA 50 μM group, 16.73% ± 2.15% for the HEA 100 μM group, and 65.54% ± 11.89% for the HEA 150 μM group. HEA enhanced the apoptotic rate in SGC-7901 and AGS cells (Figure 2C,E). Changes in fluorescence after Annexin V-FITC/PI staining showed that the 75 μM HEA group exhibited a significant increase in green fluorescence, which is a typical morphological characteristic of early apoptosis (Figure 2D). The cells treated with 150 μM HEA exhibited increased red fluorescence, indicating that the late apoptotic rate of cells increased (Figure 2D). Treatment with 75 and 150 μM HEA reduced the number of cells, suggesting that the cells may have detached from the plate. Thereafter, we detected the expression level of apoptotic key regulators. The expression level of proapoptotic protein Bax was significantly increased, and the expression of antiapoptotic regulator Bcl-2 was decreased. Moreover, HEA upregulated the expression of caspase-3, caspase-8, p53, cytochrome C, and Fas, and PARP was cleaved (Figure 2G), which may have promoted apoptosis. Pretreatment with 20 μM caspase inhibitor (Z-VAD-FMK) effectively mitigated the apoptosis induced by HEA (Figure 2F), confirming that caspases played a role in regulating HEA-induced apoptosis.

### 2.3. HEA Induced ROS Production and MMP Depolarization of Gastric Carcinoma SGC-7901 Cells

**Figure 3:**
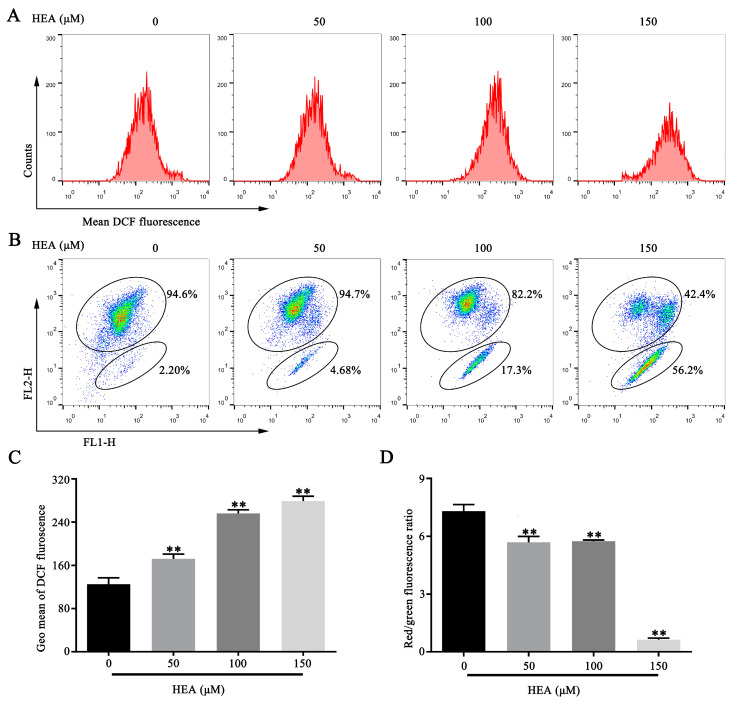
HEA induces ROS production and MMP reduction of SGC-7901 cells. (**A**) After treatment with HEA for 48 h, SGC-7901 cells were stained with ROS indicator (DCF-DA). (**B**) MMP measurement of SGC-7901 cells using JC-1 probe by flow cytometry. (**C**) Quantitation of average DCF fluorescence, indicated by Geo Mean. (**D**) Histograms of average fluorescence intensity. ** *p* < 0.01 compared with 0 μM HEA group, three repeats per group.

The production of ROS leads to mitochondrial dysfunction and DNA damage, which leads to cell apoptosis [18,19]. MMP loss is a characteristic manifestation of mitochondrial dysfunction and plays a significant role in apoptosis [20]. To determine whether the apoptosis-regulating effect of HEA was related to mitochondrial dysfunction, flow cytometry was used to measure the changes in cellular ROS and MMP after HEA treatment. The results showed that HEA could significantly increase ROS production and decrease MMP (Figure 3), suggesting that HEA-induced apoptosis may be related to mitochondrial dysfunction.

### 2.4. HEA Elevated Intracellular Ca^2+^ Levels, Leading to ER Stress-Mediated Apoptosis in Gastric Carcinoma SGC-7901 Cells

**Figure 4:**
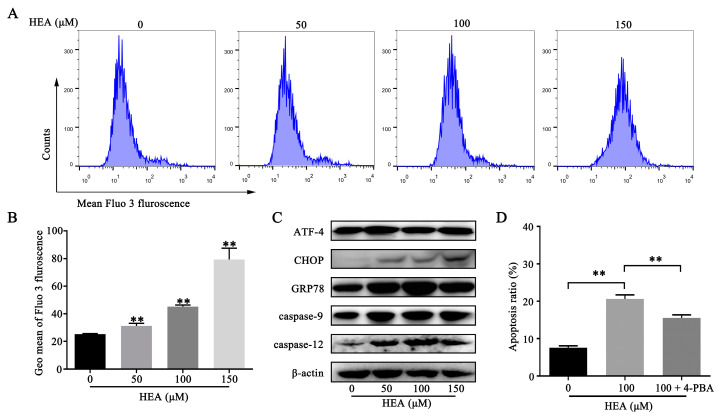
HEA-induced ER stress of SGC-7901 cells. (**A**) Cells were treated with HEA for 48 h, and intracellular Ca^2+^ was measured using Fluo-3 AM probe. (**B**) Histogram of average Fluo-3 AM, indicated by Geo Mean. (**C**) Western blot analysis of HEA on the expressions of ER stress-related proteins. (**D**) Columns for apoptotic SGC-7901 cells incubated with vehicle, HEA (100 μM), HEA (100 μM) + 4-PBA (1 mM), and ER stress inhibitor 4-PBA and HEA had those compounds added 48 h before the apoptosis detection assay. ** *p* < 0.01 compared with 0 μM HEA group, three repeats per group.

ER stress is characterized by misfolding of the ER lumen and disturbance of Ca^2+^ balance. Upregulation of ER chaperone proteins such as glucose regulatory protein 78 (GRP78) can activate the apoptotic pathways mediated by caspase-12. A Fluo-3 AM fluorescence probe was used to detect the relative changes of intracellular Ca^2+^ level after treatment with various doses of HEA in SGC-7901 cells, and Western blot analysis was used to detect the expression changes of ER stress-related proteins. HEA treatment resulted in a significant increase in fluorescence intensity, indicating a significant increase in intracellular Ca^2+^ concentration in SGC-7901 cells (Figure 4A,B). Moreover, the protein expression levels of ATF-4, CHOP, GRP78, caspase-12, and caspase-9 increased in the HEA-treated group, suggesting that HEA may have induced ER stress and activated the caspase-12-mediated apoptotic pathway (Figure 4C). The co-addition of 4-PBA (an ER stress inhibitor, 1 mM) and HEA further confirmed the role of ER stress in HEA-induced apoptosis. In SGC-7901 cells, the apoptotic rate of co-treated cells was significantly decreased compared to cells treated with HEA only, suggesting that the inhibition of ER stress weakened the apoptotic effect of HEA (Figure 4D).

### 2.5. HEA Promoted Autophagy-Mediated Apoptosis of SGC-7901 Cells

**Figure 5:**
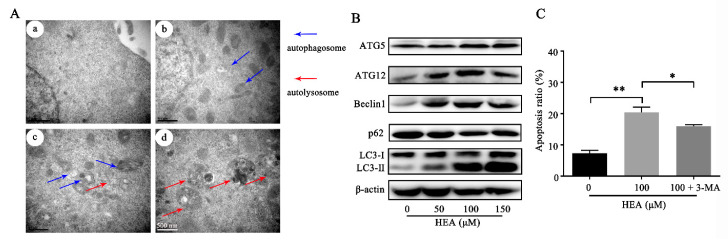
HEA-induced autophagy of SGC-7901 cells. (**A**) Representative TEM for SGC-7901 cells incubated with the following doses of HEA: (**a**) 0, (**b**) 50, (**c**) 100, and (**d**) 150 μM; blue arrows represent autophagosome, and red arrows represent autolysosome. (**B**) Expression levels of ATG5, ATG12, Beclin1, p62, and LC3 in SGC-7901 cells after treatment with HEA for 48 h by immunoblotting. (**C**) Columns for apoptotic SGC-7901 cells incubated with vehicle, HEA (100 μM), HEA (100 μM) + 3-MA (5 mM), and autophagy inhibitor 3-MA and HEA had those compounds added 48 h before the apoptosis detection assay. ** *p* < 0.01, * *p* < 0.05 compared with 0 μM HEA group, three repeats per group.

Autophagy was observed by TEM, and the expression of autophagy-related proteins was detected to evaluate whether autophagy occurred in SGC-7901 after treatment with HEA. The results of TEM showed that autophagosomes with a typical multimembrane structure appeared after HEA treatment, and autolysosome appeared in 100 μM HEA group and 150 μM HEA group (Figure 5A). Western blot analysis of autophagy-related proteins showed that the expression of LC3-II increased remarkably, and the ratio of LC3-II to LC3-I showed a tendency to increase after HEA treatment. We observed upregulations in ATG5, ATG12, and Beclin1 expressions and a downregulation in p62 expression, indicating that HEA-induced autophagy mediated apoptosis in SGC-7901 cells (Figure 5B). However, ATG12, and Beclin1 were down regulated at 150 μM, which may be related to the combination of ATG12 or Beclin1 with Bcl-2 domain to regulate apoptosis [21,22]. Moreover, the co-addition of 3-MA (an autophagy inhibitor, 5 mM) and HEA further confirmed the role of autophagy in HEA-induced apoptosis. In SGC-7901 cells, the apoptotic induction of co-treated cells was significantly lesser than for cells treated with HEA only, suggesting that the inhibition of autophagy weakened the proapoptotic effect of HEA (Figure 5C).

### 2.6. Inhibition of Tumor Growth by HEA in a Gastric Carcinoma Nude Mouse Model in Vivo

**Figure 6:**
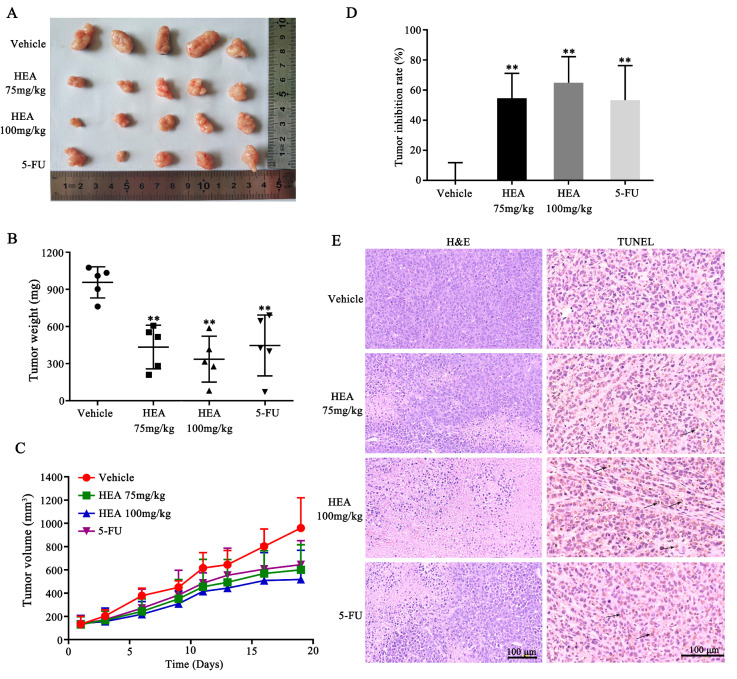
HEA inhibited tumor growth in the gastric carcinoma nude mouse model. (**A**) Morphology of the tumor xenograft resected from each nude mouse after 19 days of intragastric HEA administration. (**B**) Tumor weight of executed gastric carcinoma nude mouse model in each group. Data shown as mean ± SD. (**C**) Tumor growth curves. Data shown as mean ± SD (red circle: Vheicle, green square: HEA 75 mg/kg, blue triangle: HEA 100 mg/kg, purple triangle: 5-FU). (**D**) Tumor growth inhibition ratio (%) = (1 − tumor weight of treatment group/control group) × 100%. (**E**) H&E and TUNEL staining of xenograft tumor tissue. Black arrows indicate TUNEL-positive cells, that is, apoptotic cells. Scale bar = 100 μm. ** *p* < 0.01 compared with 0 μM HEA group, five repeats per group.

We injected female BALB/c nude mice with 2 × 10^6^ SGC-7901 cells and intragastrically administered HEA to detect the antitumor activity of HEA in vivo. Compared with the vehicle group, HEA gavage caused a reduction in tumor size (Figure 6A), tumor volume, and tumor weight in a dose-dependent manner; 75 mg/kg of HEA showed an effect similar to that of 5-FU (Figure 6B,C). After 19 days of intragastric administration, 75 mg/kg of HEA resulted in an inhibition rate of 54.66%, and 100 mg/kg of HEA exhibited an inhibition rate of 64.90% (Figure 6D). Treatment with HEA did not result in significant weight loss during the whole course of administration (Figure S2).

We performed H&E and TUNEL staining on the sections of tumors. H&E staining indicated cell death in the tumor tissues after HEA intragastric administration, wherein a clear boundary was observed between the cell injury and noninjury tissue. In addition, TUNEL staining assay showed that TUNEL-positive cells in the HEA-treated group were significantly higher compared with those of the vehicle group. Brown staining of the tumor tissue cell nuclei after TUNEL staining was observed, indicating that apoptotic cells existed (Figure 6E).

## 3. Discussion

*Cordyceps* species were verified to have antitumor activities. Both HEA and cordycepin (3ʹ-deoxyadenosine) are main components of *Cordyceps* species and are also analogues of adenosine. The antitumor effect of cordycepin has been widely reported, but studies on HEA antineoplastic activity are relatively few [6,23,24]. Previous studies have shown that EEC has an antitumor effect, and HEA is one of the main components of EEC, but no in-depth study has been conducted [13]. In this study, we demonstrated that HEA could inhibit gastric carcinoma multiplication in vivo and in vitro and explored its mechanism for the first time.

Apoptosis is the main type of cell death when DNA damage is irreparable, and inducing the apoptosis of cancer cells has become a new direction with which to improve the antitumoral effects of cancer therapy [25,26]. HEA treatment resulted in apoptosis of SGC-7901 cells; however, the apoptotic rate decreased significantly after caspase inhibitor treatment (Figure 2F). Meanwhile, the expression of caspase-3, 8, 9, and 12 increased in a dose-dependent manner (Figure 2G). HEA is speculated to induce caspase-dependent apoptosis. There are two pathways in caspase-dependent cell apoptosis: the death receptor-mediated exogenous pathway and the mitochondrial-mediated endogenous pathway [27]. The common induction of apoptosis involves one of the apoptotic modes [28,29,30]; studies on triggering endogenous and exogenous apoptosis are widely available [31,32,33]. Exogenous pathways are caspase cascades triggered by the binding of death receptor Fas or TFN-α with its extracellular ligands. This was indeed what we detected in HEA-treated cells, where Fas, caspase-3, and caspace-8 were significantly increased (Figure 2G). These findings suggested that HEA treatment stimulated exogenous apoptotic pathways. Meanwhile, mitochondria are the center of the endogenous pathway, and the changes in the permeability of the mitochondrial extracorporeal membrane lead to the release of cytochrome C, which activates caspase cascade reaction [34]. Changes in mitochondrial membrane permeability in vitro are regulated by the Bcl-2 family, and p53 can regulate the transcription of Bcl-2 family [35,36]. In this study, the expression level of p53 was elevated, leading to the upregulation of proapoptotic protein Bax and the downregulation of antiapoptotic protein Bcl-2. Moreover, the depolarization of MMP, generation of ROS, and upregulation of cytochrome C and caspase-9 confirmed that HEA treatment accords with the endogenous apoptotic pathway. After caspase cascade reaction, PARP is cut and apoptosis is induced [37]. The downregulation of PARP and the upregulation of cleaved-PARP confirmed that apoptosis triggered by HEA treatment was involved in the two pathways of caspase-dependent apoptosis.

To bring insight into the mechanism of tumor suppression by HEA, especially in apoptosis, we examined potential targets. The flow cytometry, protein-level detection and transcriptomics showed the consistent tendency that the antitumor mechanisms of HEA were involved in ROS production, MMP depolarization, ER stress changes, and autophagy. These results reflected that HEA affected the mitochondrial homeostasis of gastric carcinoma cells. In fact, there are more and more anti-tumor studies involving multiple death pathways. Pinocembrin can induce ER stress and mitochondria-mediated apoptosis, and suppress autophagy in melanoma [38]. Oxaliplatin induces cell death via ER stress, autophagy, and ROS production in Caco-2 cells [39]. Shikonin induces apoptosis, G2/M phase arrest, and autophagy in A375 cells via the activation of ROS-mediated ER stress and p38 pathways [40]. Interaction and influence of multiple processes cause cell death. ER stress can lead to apoptosis and autophagy [41,42]. Autophagy can suppress the induction of apoptosis by inhibiting the activation of apoptosis-related caspase, thereby reducing cell damage. It can also induce apoptosis under certain circumstances. Furthermore, the activation of apoptosis-related proteins can also inhibit autophagy by degrading autophagy-related proteins [43]. In this study, both ER stress inhibitor and autophagy inhibitor decreased the apoptotic level, which confirmed that ER stress and autophagy were the two participants in HEA-induced apoptosis of gastric carcinoma cells (Figure 4D and Figure 5C). The essence of multiprocess interaction and influence is the interaction between signal factors and signal pathways. ROS and Ca^2+^ overload can lead to increased MMP [44]. As one of the intensively studied oncogenes, p53 participates in cell apoptosis and cycle regulation and also activates AMPK to promote autophagy [45,46]. ER stress-induced autophagy and apoptosis also share a common upstream signaling pathway, the IRE1–JNK pathway [43]. AKT kinase phosphorylates Bcl-2 family member Bad, thereby suppressing apoptosis [47]. Moreover, AKT can also negatively regulate cell cycle through the p53/p21-dependent pathway [48]. P62 is a well-known autophagic protein, which can bind to autophagic receptor protein Atg8/LC3 and then affect cell autophagy. It can also trigger caspase cascade reaction by activating caspase-8 to mediate apoptosis [49]. In this research, the interactions of signaling factors among ER stress-mediated and autophagy-mediated apoptosis deserves further study.

## 4. Materials and Methods

### 4.1. Reagents

DMEM and 0.25% Trypsin were purchased from HyClone (Logan, UT, USA). HEA was purchased from Solarbio (Beijing, China). FBS was obtained from Gibco (Grand Island, NY, USA). 5-FU was obtained from Sigma-Aldrich (St. Louis, MO, USA). The Annexin V-FITC/PI Apoptosis Analysis Kit was obtained from Tianjin Sungene (Tianjin, China). The Mitochondrial Membrane Potential (MMP) assay kit with JC-1, Cell Counting Kit-8 (CCK-8), Reactive Oxygen Species (ROS) assay kit, and Fluo-3 AM fluorescent probes were purchased from Beyotime (Jiangsu, China). Sodium phenylbutyrate (4-PBA) and 3-methyladenine (3-MA) were purchased from Macklin (Shanghai, China).

### 4.2. Cell Culture

Human embryonic kidney 293 (HEK293) cells, human gastric carcinoma SGC-7901 cells were purchased from Beyotime (Jiangsu, China), human gastric carcinoma AGS cells was obtained from the Cell Research Institute of the Chinese Academy of Sciences (Shanghai, China). Cells were authenticated by STR analysis and cultured in complete medium (DMEM medium containing 10% FBS) under a humid 5% CO_2_ atmosphere at 37 °C.

### 4.3. Cytotoxicity Assay In Vitro

Cytotoxicity assay in vitro was performed using CCK-8 in accordance with the manufacturer’s instructions. Cells were seeded in 96-well plates (Costar Corning, Rochester, NY, USA) at a density of 5 × 10^3^ cells/well. After 24 h, 200 μL of DMEM medium-soluble HEA with varying dosages were added to each well, every concentration repeated in five wells. After 48 h, the cells were incubated with 10 μL CCK-8 at 37 °C for 2 h. The absorbance was measured at 450 nm (A_450_) by a multimode reader (Thermo Electron Corporation, MA, USA).

### 4.4. Morphological Observations

Cells were seeded in six-well plates and then treated with varying HEA concentrations. After 48 h, cell morphology was analyzed using an Olympus phase-contrast microscope (Olympus, Tokyo, Japan) under 20× magnification.

### 4.5. Assay of Cell Apoptosis

SGC-7901 and AGS cells were seeded in six-well plates with 1 × 10^5^ cells/well and then treated with varying HEA concentrations after 24 h. After 48 h of incubation, cells were harvested and rinsed and stained with Annexin V-FITC and PI. The apoptotic rate was detected by BD FACSVerse flow cytometer as previously mentioned [50]. The stained cells were observed by confocal laser scanning microscopy.

### 4.6. Determination of Endogenous ROS

We performed flow cytometry assay using 2′,7′-dichlorofluorescein-diacetate (DCFH-DA) staining to determine cellular ROS as previously described [51]. Briefly, after treatment with HEA, the cells were harvested, rinsed thrice, and incubated with 1 mL DCFH-DA (10 μM) working solution for 20 min at 37 °C. The cells were washed thrice with DMEM to remove unbound DCFH-DA and analyzed by flow cytometry.

### 4.7. Detection of MMP

MMP level was detected by JC-1 probe assay kit. In cells with high MMP, J-aggregates produced red fluorescence, whereas in cells with low MMP, JC-1 monomer produced green fluorescence. The proportion of mitochondrial depolarization was measured by the ratio of red to green fluorescence. In brief, after treatment with HEA, the cells were collected and cultured with JC-1 probe (20 μg/mL) for 30 min at 37 °C. Changes in MMP were analyzed by flow cytometry.

### 4.8. Intracellular Ca^2+^ Level Detection

Intracellular Ca^2+^ was detected by Fluo-3 AM probe assay kit according to the protocol. In general, after treatment with HEA, SGC-7901 cells were collected and incubated with Fluo-3 AM probe (5 μM) for 30 min at 37 °C. Changes in intracellular Ca^2+^ were analyzed using a flow cytometer.

### 4.9. Transmission Electron Microscopy (TEM)

SGC-7901 cells were treated with various doses of HEA for 48 h and then collected and immobilized in 2.5% glutaric dialdehyde at 4 °C overnight. After removing 2.5% glutaric dialdehyde, the samples were washed with PBS thrice and fixed with 1% H_2_[OsO_4_(OH)_2_] for 2 h. Thereafter, the samples were rinsed with PBS thrice, followed by gradient dehydration with 30%, 50%, 70%, 80%, 90%, 95%, and 100% ethanol and acetone successively with 15 min at every turn. The samples were treated with Spurr embedding agent (Sigma-Aldrich, St. Louis, MO, USA) and sliced by an ultrathin slicer (Leica UC7, Wetzlar, Germany). The sections were stained in lead citrate solution and 50% ethanol saturated solution of uranium hydrogen acetate successively and observed under a TEM (Hitachi H-7650, Tokyo, Japan).

### 4.10. Western Blot Analysis

After 48 h of HEA treatment, SGC-7901 cells were collected, total protein was extracted, and Western blot analysis was performed as previously described [13]. Primary rabbit antibodies against β-actin (1:3000), AIF (1:2000), Bcl-2 (1:2000), and Bax (1:2000) were purchased from HUABIO (Hangzhou, China). Primary antibodies against caspase-3 (1:2000), caspase-8 (1:2000), caspase-9 (1:2000), caspase-12 (1:1000), cytochrome C (1:500), poly(ADP-ribose) polymerase (PARP) (1:2000), cleaved-PARP (1:2000), and p53 (1:1000), were purchased from Cell Signaling Technology (Beverly, MA, USA). Primary antibodies against ATF4 (1:1000), CHOP (1:500), p62 (1:1000), and Beclin1 (1:1000) were obtained from Proteintech (Rosemont, IL, USA). Primary antibodies against LC3 (1:500), ATG5 (1:1000), and ATG12 (1:1000) were obtained from MBL (Nagoya, Japan). Anti-rabbit lgG horse-radish peroxidase-conjugated secondary antibody (1:3000) was purchased from Abcam (Cambridge, MA, USA).

### 4.11. In Vivo Efficacy of HEA

Female BALB/c nude mice (16–18 g body weight) aged 4–5 weeks were purchased from Shanghai SLAC Laboratory Animal Co., Ltd. (Shanghai, China) with production license number SCXK 2017-0005 and certificate number 20170005006588. Mice were handled in accordance with the guidelines of the National Institutes of Health (NIH). The mice were housed at the Laboratory Animal Center of Hangzhou Normal University and maintained in an SPF condition, in accordance with the animal care and use guidelines of the Organizational Animal Care and Use Committee. Approximately 2 × 10^6^ cells of SGC-7901 were injected subcutaneously to establish transplanted tumor in nude mice. Two weeks after SGC-7901 cells injection, the nude mice with tumor volume of 100–150 mm^3^ were randomly placed into four groups (5 mice/group): the vehicle group (PBS), HEA-75 mg/kg group, HEA-100 mg/kg group, and 5-FU (25 mg/kg) group. During the study, body weight and tumor volume were monitored twice a week in nude mice. Tumor volume was measured using a Vernier caliper and calculated using the formula V = 1/2 (length × width^2^). Each group of mice received intragastric treatment of drugs daily. After 19 days, the mice were killed via cervical dislocation, and tumors were dissected and weighted. Tumor tissue was immobilized in 4% paraformaldehyde and stained with hematoxylin and eosin (H&E) and TUNEL.

### 4.12. Statistics

SPSS software 17.0 (SPSS Inc., Chicago, IL, USA) and GraphPad Prism 6.0 (GraphPad, San Diego, CA, USA) were used for statistical analysis. One-way ANOVA and t-tests were used to evaluate the statistical significance. Data were expressed as means ± SDs.

## 5. Conclusions

This study proved for the first time that HEA had anti-gastric carcinoma activity in vitro and in vivo. HEA played an anti-tumor role by promoting apoptosis of human gastric carcinoma cells in vitro. Further studies showed that HEA could induce ROS production and MMP depolarization to promote the apoptosis of gastric carcinoma cells. Meanwhile, we found that HEA-induced autophagy and ER stress also played proapoptotic roles. In vivo studies also revealed that HEA could also significantly inhibit the growth of tumors in nude mice and induce the apoptosis of tumor tissues. These results together demonstrated the anticancer effect of HEA, suggesting that HEA is a promising chemotherapeutic agent for gastric carcinoma.

## Supplementary Materials

Supplementary materials can be found at https://www.mdpi.com/1422-0067/21/16/5815/s1.

## Abbreviations

3-MA	3-methyladenine
4-PBA	Sodium phenylbutyrate
CCK-8	Cell Counting Kit-8
DCFH-DA	2′,7′-dichlorofluorescein-diacetate
DMEM	Dulbecco’s modified Eagle’s medium
EEC	Ethanolic extract of *Cordyceps cicadae*
ER	Endoplasmic reticulum; FBS, Fetal bovine serum
FITC	Fluorescein isothiocyanate
HEA	N6-(2-Hydroxyethyl)-adenosine
H&E	Hematoxylin-eosin
JC-1	5,5′,6,6′-Tetrachloro-1,1′,3,3′-tetraethyl-imidacarbocyanine iodide
MMP	Mitochondrial membrane potential
PI	Propidium iodide
ROS	Reactive oxygen species
TEM	Transmission electron microscopy
