# Supplementary material for: N6-(2-hydroxyethyl)-Adenosine Induces Apoptosis via ER Stress and Autophagy of Gastric Carcinoma Cells In Vitro and In Vivo
Source: Int J Mol Sci. 2020 Aug 13;21(16):5815. doi: 10.3390/ijms21165815 (PMC7461581; doi:10.3390/ijms21165815)
Supplement: Supplementary file 1 [file ijms-21-05815-s001.pdf]

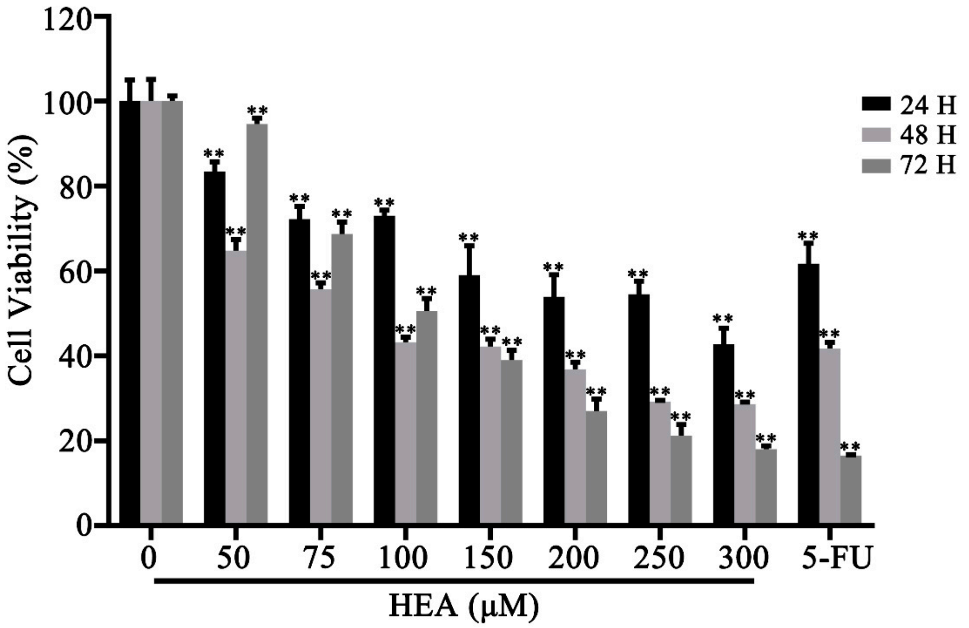

Figure. S1. Evaluation of growth inhibition CCK-8 assay by various doses of HEA at different treatment time in AGS cells, ** p < 0.01compared with 0 μM HEA group.

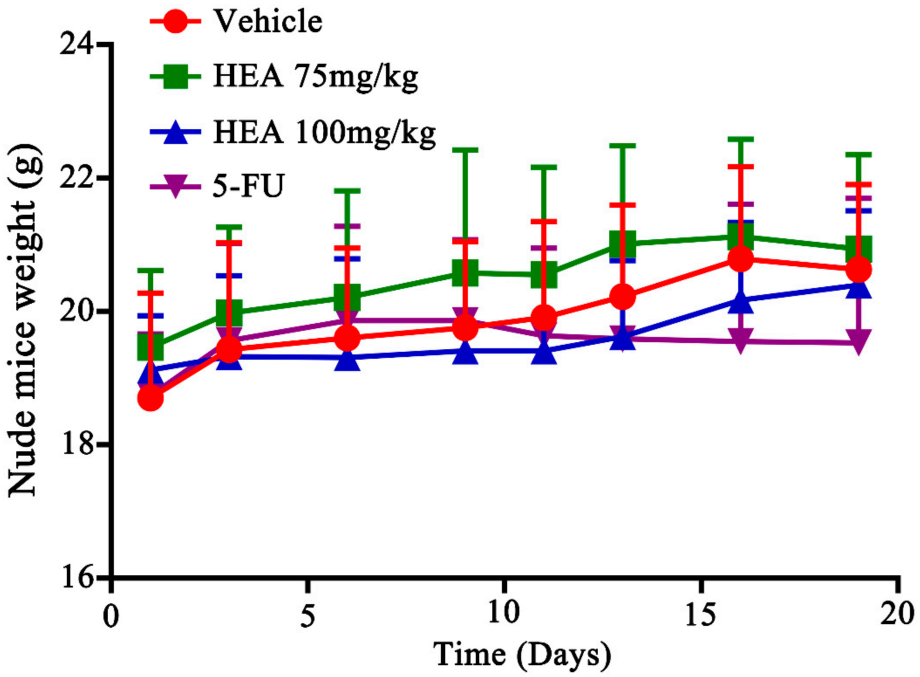

Figure. S2. Weight changes of gastric carcinoma nude mice in each group. Data showed as Mean + SD
